# Function of Adipose-Derived Mesenchymal Stem Cells in Monocrotaline-Induced Pulmonary Arterial Hypertension through miR-191 via Regulation of BMPR2

**DOI:** 10.1155/2019/2858750

**Published:** 2019-04-16

**Authors:** Caixin Zhang, Pengbo Wang, Anaz Mohammed, Zhewen Zhou, Shuwen Zhang, Songshi Ni, Zhiyuan Tang

**Affiliations:** ^1^Department of Respiratory and Critical Care Medicine, Affiliated Hospital of Nantong University, Nantong, 226001, Jiangsu, China; ^2^Department of Dermatology, Affiliated Hospital of Nantong University, Nantong, 226001, Jiangsu, China

## Abstract

Pulmonary arterial hypertension (PAH) is a serious condition. However, prevailing therapeutic strategies are not effective enough to treat PAH. Therefore, finding an effective therapy is clearly warranted. Adipose-derived mesenchymal stem cells (ASCs) and ASCs-derived exosomes (ASCs-Exos) exert protective effects in PAH, but the underlying mechanism remains unclear. Using a coculture of ASCs and monocrotaline pyrrole (MCTP)-treated human pulmonary artery endothelial cells (HPAECs), we demonstrated that ASCs increased cell proliferation in MCTP-treated HPAECs. Results showed that ASCs-Exos improved proliferation of both control HPAECs and MCTP-treated HPAECs. In addition, by transfecting ASCs with antagomir we observed that low exosomal miR-191 expression inhibited HPAECs proliferation whereas the agomir improved. Similar results were observed in vivo using a monocrotaline (MCT)-induced PAH rat model following ASCs transplantation. And ASCs transplantation attenuated MCT-induced PAH albeit less than the antagomir treated group. Finally, we found that miR-191 repressed the expression of bone morphogenetic protein receptor 2 (BMPR2) in HPAECs and PAH rats. Thus, we conjectured that miR-191, in ASCs and ASCs-Exos, plays an important role in PAH via regulation of BMPR2. These findings are expected to contribute to promising therapeutic strategies for treating PAH in the future.

## 1. Introduction

It is considered to be pulmonary arterial hypertension (PAH) when the mean pulmonary artery pressure (mPAP) is above 25 mmHg at rest [1]. As the disease progresses, pulmonary vascular resistance increases, leading to right heart failure and mortality [2]. Vascular remodeling is the key pathological feature of PAH, characterized by endothelial dysfunction, activation of fibroblasts, and smooth muscle cells. Endothelial dysfunction included suppression of normal apoptosis and excessive proliferation of endothelial cells [3]. So far, the modern therapy has improved clinical state and extended life by a few years, but the vascular changes remain progressive [4]. And no effective therapy that can either terminate or reverse the hyperplasia of human pulmonary artery endothelial cells (HPAECs) is available.

Mesenchymal stem cells (MSCs) are major members of stem cells that were first discovered in bone marrow. Human adipose is an abundant and accessible source of adipose-derived mesenchymal stem cells (ASCs) [5]. ASCs are capable of reducing inflammation in damaged tissues, improving angiogenesis, and reducing apoptosis due to their paracrine secretory potential, mitochondrial transfer, and secretion of exosomes [6–9]. Previous studies have shown that MSCs can relieve the development of PAH in animal models [10, 11]. However, no relevant study has been carried out to explore the contribution of ASCs in monocrotaline pyrrole (MCTP)-treated HPAECs and monocrotaline (MCT)-induced PAH rat model.

Exosomes are a type of extracellular vesicles between 50 and 150 nm in size, secreted by almost all types of cells, which are widely present in various body fluids [12]. Exosomes are vesicles encapsulated by a lipid bilayer containing biologically active substances such as proteins, microRNAs (miRNAs), long noncoding RNAs (lncRNAs), and rRNAs [13–15]. Recent studies have found that ASCs-derived exosomes (ASCs-Exos) can mimic biological functions of mother cells. ASCs-Exos are being used to alleviate tissue damage and treat incurable diseases, which has broad prospects for development [16–18]. However, the effects of ASCs-Exos on HPAECs and the underlying mechanisms are rarely investigated.

MicroRNAs (miRNAs) belong to a class of endogenous single-stranded, noncoding RNAs of approximately 22 nucleotides that repress mRNA translation or stability by binding to the 3′ UTR of the target gene. In mammals, miRNAs are involved in more than 60% of all protein-coding processes [19, 20]. Among the various RNAs present in exosomes, miRNAs constitute 76.2% of the total RNAs, and miR-191 is one of the most abundant miRNAs in ASCs-Exos [21]. A recent study has shown elevated miR-191 in the circulation of PAH patients [22]. However, its function in PAH remains obscure. In this study, we aimed to investigate the effects of exosomal miR-191 on the growth of MCTP-treated HPAECs as well as the underlying mechanism. Furthermore, we also explored the function of ASCs transfected with miR-191 in the development of PAH in MCT-induced PAH rats.

## 2. Materials and Methods

### 2.1. Reagents and Antibodies

The antibodies of CD9, CD63, CD81, and bone morphogenetic protein receptor 2 (BMPR2) were obtained from Abcam (Cambridge, MA, USA). Monocrotaline was purchased from the Fluorochem (Hadfield, Derbyshire, UK). The agomir and antagomir were obtained from Biomics Biotech (Nantong, China).

### 2.2. Animals

Thirty-five male Sprague-Dawley rats (240 ± 30 g) were provided by the Laboratory Animal Center of Nantong University. All rats were fed in a well-ventilated room with 12-hour light and dark cycle, with standard water and food ad libitum. All courses followed the Guidelines of Chinese Council on Animal Research and the Guidelines of Animal Care to minimize the suffering of animals. This study has been approved by the Administration Committee of Experimental Animals, Jiangsu Province, China (Approval ID: SYXK (SU) 2012-0031).

### 2.3. Isolation and Culture of Cells

Human ASCs (hASCs) were isolated and cultured as described by Bradley et al. [23]. Briefly, the adipose tissue was collected from a 25-year-old male's abdomen, washed 3 times with phosphate buffered saline (PBS) (Hyclone, Utah, USA). This work was approved by Ethics Committee of Affiliated Hospital of Nantong University (Examination number: 2018-K020). The fascia and small blood vessels were removed and moistened with 1% Collagenase I (Biosharp, Hefei, China) for 2 hours; the stem cells were then placed in culture flasks with DMEM/High Glucose medium (Hyclone) supplemented with 10% fetal bovine serum (Gibco, Carlabad, USA) and incubated at 37°C. Initially the medium was changed after 72 hours, and thereafter the medium was changed every 2 days. FACS was used to characterize the ASCs. Osteogenic differentiation and adipogenic differentiation assays were performed by the cells of passage 4. ASCs from passages 3-5 were used for the subsequent experiments.

HPAECs were purchased from BeNa Culture Collection (BeNa, Beijing, China). The cells were maintained in DMEM/High Glucose medium supplemented with 10% fetal bovine serum at 37°C in a humidified chamber, 5% CO_2_, 95% air atmosphere.

### 2.4. Isolation and Characterization of ASCs-Exos

First, ASCs were cultured in medium supplemented with 10% serum for 24 hours. Then serum-free medium was used instead, and the cells were incubated for another 24 hours. Exosomes were collected from each conditioned medium by ultracentrifugation at 150,000g for 2 hours at 4°C (CS150GXL, Hitachi, Japan). Then exosomes were washed once by PBS and they were ultracentrifuged again. Exosomes were suspended in 100 *μ*l PBS. Twenty microliters exosomes were loaded onto a formvar/carbon-coated grid, negatively stained with 3% aqueous phosphotungstic acid for 1 minute and observed by transmission electron microscope (TEM, JEM-1230, JEOL, Tokyo, Japan). BCA Protein Assay Kit (Biosharp) was used to assess the exosomes protein concentration.

### 2.5. Exosomes Labeling

For the exosome-labeling experiments, purified exosomes were fluorescently labeled using PKH67 Green Fluorescent Cell Linker Mini Kit (Sigma-Aldrich, USA). Simply, labeled exosomes were collected by ultracentrifugation as described above and resuspended in PBS. HPAECs were seeded and incubated for 1 hour with PKH67-labeled exosomes. Then, the cells were fixed with 4% paraformaldehyde and washed 3 times with PBS. Nuclei were incubated with DAPI (Beyotime, Shanghai, China) for 10 minutes and washed 3 times with PBS. Images were captured by the Olympus Microscope (Olympus, Japan).

### 2.6. Preparation of MCTP

MCT was converted to MCTP using tetrabromo-1,2-benzoquinone (Sigma-Aldrich) [24]. The product was stored in N, N-dimethylformamide (DMF) at −20°C prior to use.

### 2.7. Cell Viability Assay

Cell viability was measured by CCK-8 assay (Beyotime). HPAECs were prepared in 96-well cell plates. After 24-hour of incubation, the culture medium was removed and HPAECs were incubated with various concentrations of MCTP (0-120 *μ*g/ml) or vehicle (DMF with the identical dilution rate as MCTP) for different time (0-72h). 10 *μ*l CCK-8 solution was added to each well, and the viability was measured at 450 nm by an enzyme linked immunosorbent assay reader (Bio Tek, Winooski, USA).

### 2.8. Overexpression and Knockdown of miR-191 in ASCs and ASCs-Exos

ASCs were starved in serum-free medium for 24 hours and then transfected with miR-191 agomir (50 nM), miR-191 antagomir (100 nM), agomir negative control (agomir NC) (50 nM), and antagomir negative control (antagomir NC) (50 nM), respectively, using Lipofectamine 2000 (Thermo Fisher, Carlsbad, USA) transfection reagent according to the manufacturer's instructions. Twenty-four hours later, serum-free medium was replaced by completed medium. Then exosomes were collected by the method mentioned above, and RT-PCR was used to verify the expression of miR-191. The agomir and antagomir sequences used are shown in Table 1.

### 2.9. RT-PCR Assay

Total RNA was extracted from cells by TRIzol Reagent (Ambion, Carlsbad, USA) and reverse-transcripted by a Reverse Transcriptase kit (Thermo Fisher) according to the manufacturer's protocol. The transcripts were quantified with SYBR Green (Vazyme, Nanjing, China) on a qTOWER 2.2 RT-PCR apparatus (Analytik Jena, Jena, Germany). The relative levels of cellular miRNA were quantified with the 2^−ΔΔCt^ after normalizing to U6. The primers used for RT-PCR were designed by RiboBio (Guangzhou, China): Bulge-Loop™ miRNA qRT-PCR Primer Sets.

### 2.10. Coculture of ASCs and HPAECs

Coculture was performed using transwell chambers with 3.0-*μ*m pore size. The upper chamber was seeded with 1 × 10^5^ ASCs, while 1 × 10^4^ HPAECs were seeded in the lower chamber. Twenty-four hours later, HPAECs were fixed in methanol for 10 minutes, stained with crystal violet for another 15 minutes, then washed 3 times with PBS. Digital images were photographed under a microscope.

### 2.11. Western Blot

The protein extracts were obtained by using RIPA buffer (Beyotime), and protein concentration was determined by the BCA kit. The membrane was blocked with 5% fat-free milk (Yili, Hohhot, China) for 2 hours at room temperature (RT) and then incubated with the following primary antibodies at 4°C overnight. The dilutions of antibodies were anti-CD9 (1:1000), anti-CD63 (1:500), anti-CD81 (1:1000), and anti-BMPR2 (1:500). Samples were normalized to GAPDH (1:3000; Abways, Shanghai, China). The secondary antibodies were incubated for 2 hours at RT. Then protein bands were detected by ChemiDoc XRS + system (Bio-Rad, USA).

### 2.12. Rat PAH Model and ASCs Injection

Rats were given a single intraperitoneal injection of MCT (60mg/kg) to induce PAH, and the control group was treated with saline. After two weeks, the seven groups of rats were treated as follows: none (control group), saline (MCT group), ASCs (MCT + ASC group), miR-191 agomir transfected stem cells (MCT + agomir group), miR-191 antagomir transfected stem cells (MCT + antagomir group), agomir NC cells (MCT + agomir NC group), and antagomir NC cells (MCT + antagomir NC group). The number of cells injected was 3 × 10^6^. At the end of the fourth week, all rats followed right ventricle intubation and the right ventricular systolic pressure (RVSP) was measured.

### 2.13. Measurement of RVSP, Right Ventricle Hypertrophy

All rats were anesthetized with 10% chloral hydrate (30 ml/kg, i.p.). Rats were kept in a supine position, the right jugular vein was isolated, and a PE-50 polyethylene tube filled with heparin saline was inserted into the jugular vein. The catheter was then further inserted into the right ventricle, and systolic pressure was recorded by the BIOPAC multi-lead physiography (BIOPAC Systems, Santa Barbara, USA). Then the hearts were collected, and the right ventricle and left ventricle + septum were carefully separated and weighted. The weight ratio of RV/LV + S was calculated.

### 2.14. Vascular Morphology of Distal Pulmonary Arteries

The left upper lobe was obtained from each group and fixed with 10% formalin. H&E staining was used for histological observations. Six images of pulmonary artery (external diameter 50~200 *μ*m) of each group were randomly captured and analyzed to evaluate the remodeling of pulmonary artery. CSA and MT + IT were measured by Image-Pro Plus software.

### 2.15. Immunohistochemistry

Two weeks after ASCs transplantation, heart and lung tissues were collected and fixed in 4% paraformaldehyde. The primary antibodies were applied to incubate the sections overnight at 4°C. The secondary antibody was used to cover the tissues for 50 minutes at RT. Immunopositive cells were counted using Image-Pro Plus software. The dilution of antibody was anti-BMPR2 (1:200).

### 2.16. Statistical Analysis

All experiments were conducted independently at least three times. All data were expressed as the mean ± SD. Statistical analysis was performed using the GraphPad Prism 6 software. We used Student's t-test to analyze the statistical significance of differences between two groups, and comparisons among three or more groups were made with one-way ANOVA analysis. P value < 0.05 was considered statistically significant.

## 3. Results

### 3.1. Characterization of ASCs Isolated from Adipose Tissue

**Figure 1:**
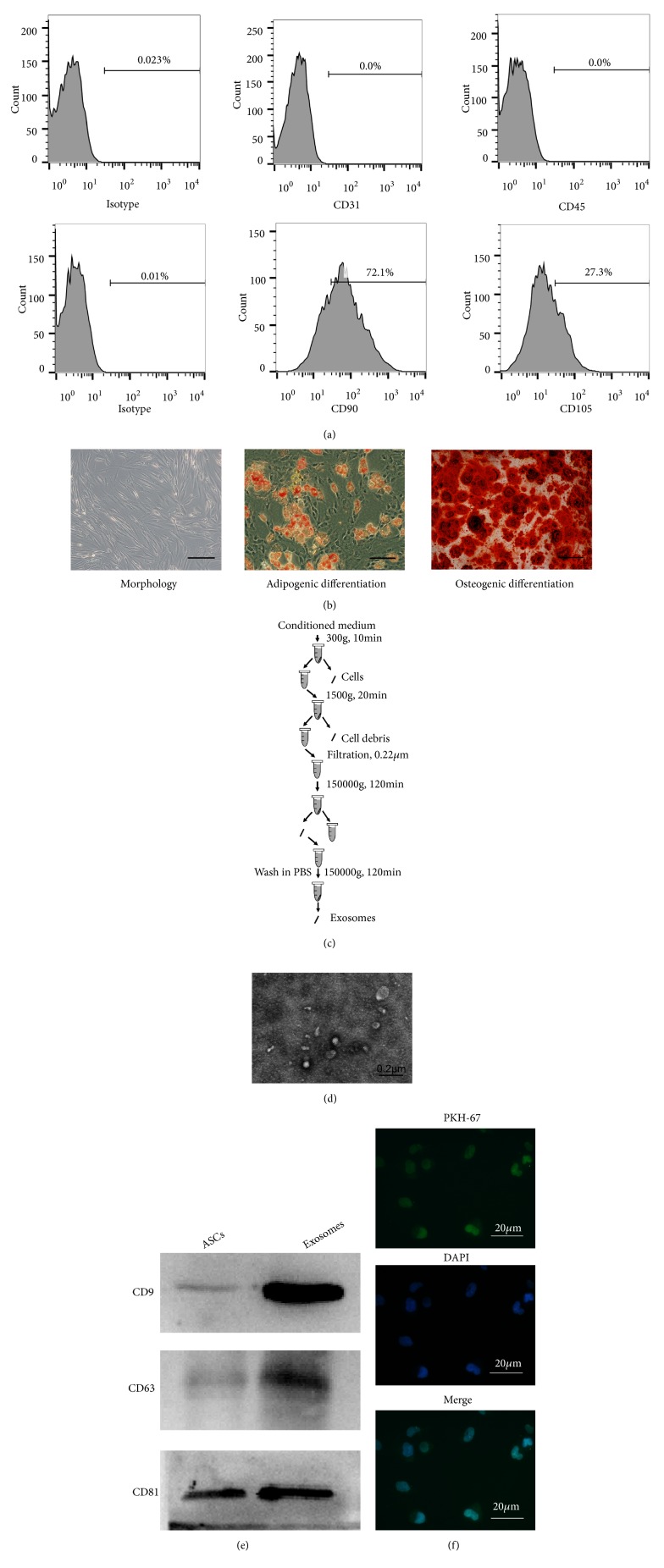
Characterization of ASCs and ASCs-Exos. (a) The expression level of CD31, CD45, CD90, and CD105 in ASCs was detected by FACS. (b) Morphology, adipogenic differentiation, and osteogenic differentiation of ASCs cultured in vitro. Scale bars = 100 *μ*m. (c) Scheme of exosomes isolation by ultracentrifugation. (d) Representative electron microscopy image of ASCs-Exos. Scale bars = 0.2 *μ*m. (e) Western blot analysis of exosomal markers. ASCs protein was used as control for exosomes characterized. (f) ASCs-Exos were labeled with PKH-67. ASCs-Exos transferring into HPAECs were tracked using a fluorescence microscope. Scale bars = 20 *μ*m.

Fluorescence-activated cell sorting (FACS) analysis showed that cell surface markers CD90 and CD105 were highly expressed while CD31 and CD45 staining was not observed in ASCs (Figure 1(a)). Morphology, adipogenic differentiation, and osteogenic differentiation are shown in Figure 1(b). As expected, at passage 3 most ASCs were adherent and spindle-like after culturing for 7 days. In addition, ASCs formed alizarin-red positive mineral deposits and adipogenic potential. Therefore, all data suggested successful isolation and characterization of ASCs from adipose tissue.

### 3.2. ASCs Improved HPAECs Proliferation

**Figure 2:**
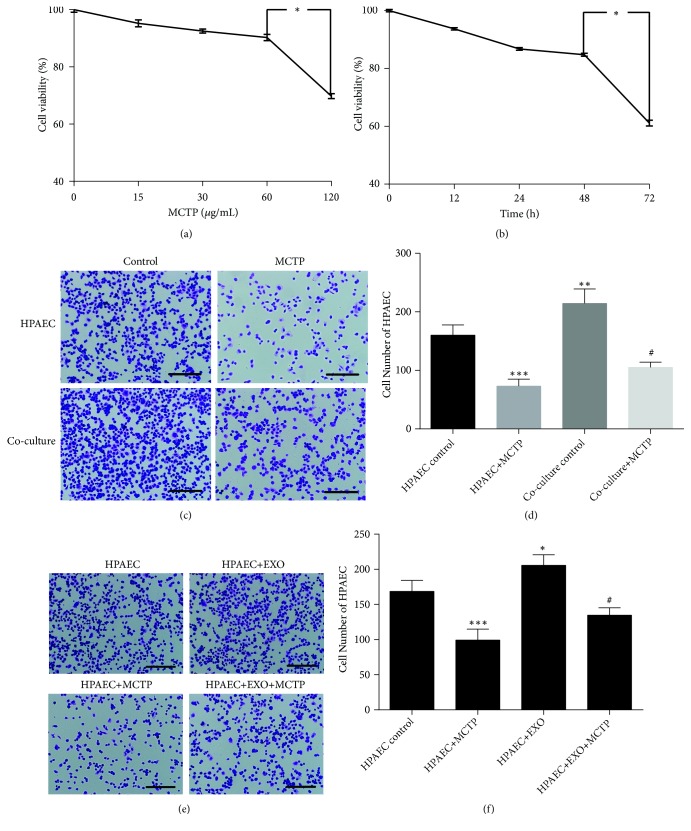
ASCs and ASCs-Exos improved HPAECs proliferation. (a) Cell viability was analyzed by using the CCK8 assay after exposure to MCTP at concentrations of 0, 15, 30, 60, and 120 *μ*M for 24 h. (b) Cell incubation with MCTP for 0, 12, 24, 48, and 72 h at the concentration of 60 *μ*M. (c) Responding pictures of HPAECs cocultured with or without ASCs. Scale bars = 100 *μ*m. (d) The number of HPAECs, counted in 3 fields under × 10 objective lens. Data are presented as mean ± SD, n =3. *∗∗*P < 0.01, *∗∗∗*P < 0.001 vs. HPAECs control group, ^#^P < 0.05 vs. coculture control group. (e) Representative pictures of HPAECs cultured with or without exosomes. Scale bars = 100 *μ*m. (f) The number of HPAECs cultured with or without exosomes, counted in 3 fields under × 10 objective lens. Data are presented as mean ± SD, n =3. *∗*P < 0.05, *∗∗∗*P < 0.001 vs. HPAECs group, ^#^P < 0.05 vs. HPAECs+MCTP group.

Viability of HPAECs incubated with different concentrations of MCTP was assessed by CCK8 assay. Based on significant differences, we selected 60 *μ*M MCTP (P < 0.05) for 48 h (P < 0.05) to treat HPAECs (Figures 2(a) and 2(b)). HPAECs were cocultured with or without ASCs to determine the effect of ASCs on HPAECs. Proliferation of HPAECs decreased significantly after incubating with 60 *μ*g/ml MCTP. However, when cocultured with ASCs, growth of HPAECs was significantly increased (Figures 2(c) and 2(d)). Therefore, we hypothesized that ASCs produce bioactive factors that influence the proliferation of HPAECs.

### 3.3. ASCs-Exos Improved Cell Proliferation in MCTP-Induced HPAECs

Exosomes were isolated using ultracentrifugation (Figure 1(c)) and identified using transmission electron microscope. We confirmed heterogeneous lipid bilayered vesicles, approximately 50-100 nm in diameter (Figure 1(d)). Western blot analysis showed increased expression of exosomal surface markers, CD63, CD9, and CD 81, compared with whole ASCs lysates (Figure 1(e)). HPAECs cocultured with PKH-67-labeled exosomes for 1 hour exhibited high uptake efficiency as detected by fluorescence microscopy (Figure 1(f)). Results, thus, indicated successful isolation of exosomes and their uptake by HPAECs.

To further evaluate the effect of exosomes on HPAECs, HPAECs were cultured with or without exosomes. As shown in Figures 2(e) and 2(f), exosomes improved proliferation of both HPAECs and MCTP-treated HPAECs. Results showing that ASCs-Exos modulated HPAECs proliferation, in turn, supported our conjecture that ASCs secreted certain bioactive factors to affect HPAECs proliferation.

### 3.4. Circulating Exosomes Containing miR-191 Affected Proliferation by Targeting BMPR2

**Figure 3:**
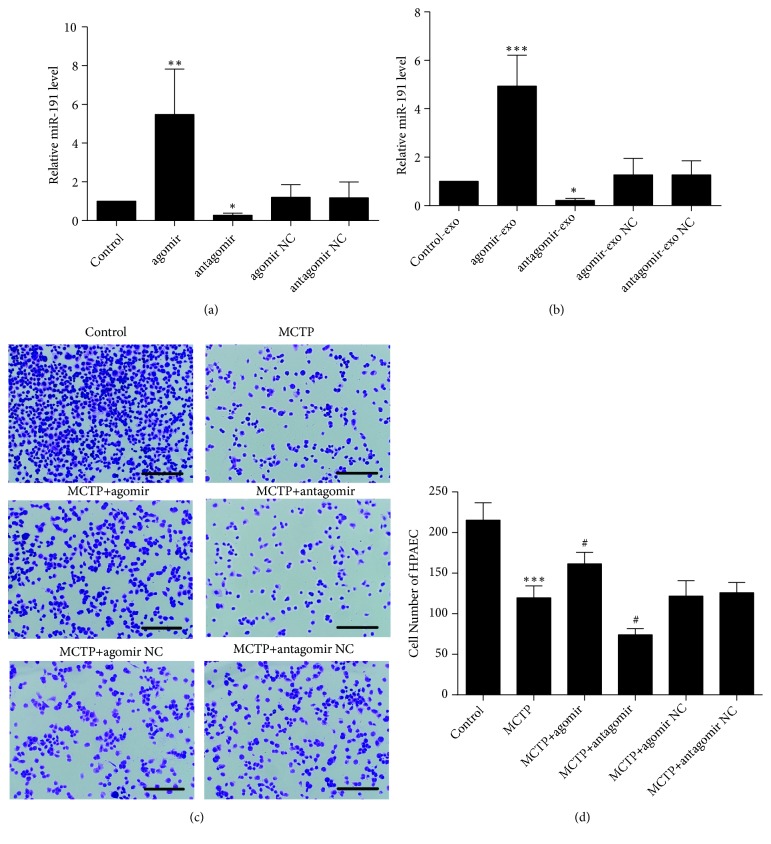
ASCs-Exos containing miR-191 affected HPAECs proliferation. (a, b) The expression levels of miR-191 in ASCs and exosomes. Data was presented as the mean ± SD, n =3. *∗*P < 0.05, *∗∗*P < 0.01 vs. control group. (c) Representative pictures of HPAECs cultured with exosomes that ASCs transfected with agomir or antagomir. Scale bars = 100 *μ*m. (d) The number of HPAECs in (c), counted in 3 fields under × 10 objective lens. Data are presented as mean ± SD, n =3. *∗∗∗*P < 0.001 vs. control group, ^#^P < 0.05 vs. MCTP group.

Real-time PCR (RT-PCR) was performed to quantify the relative levels of miR-191 in ASCs and exosomes after transfection of miR-191, showing that miR-191 was enriched in ASCs and ASCs-Exos after transfection with agomir; meanwhile, miR-191 was poorly expressed after transfection with antagomir (Figures 3(a) and 3(b)). Next, we found that high exosomal miR-191 markedly accelerated HPAECs proliferation, whereas low exosomal miR-191 attenuated them (Figures 3(c) and 3(d)). These results collectively suggested that exosomes expressing different levels of miR-191 regulate HPAECs proliferation.

Posttranscriptional gene regulation is the major biological function of miRNA. In order to identify downstream targets of miR-191, we investigated BMPR2 expression in HPAECs. Expression of BMPR2 was analyzed by western blotting (Figure 5(a)). The result suggested that expression of BMPR2 protein in HPAECs was remarkably increased in the antagomir group and decreased in the MCT and agomir groups.

### 3.5. ASCs Ameliorated Pulmonary and Heart Function in a Rat PAH Model through miR-191

**Figure 4:**
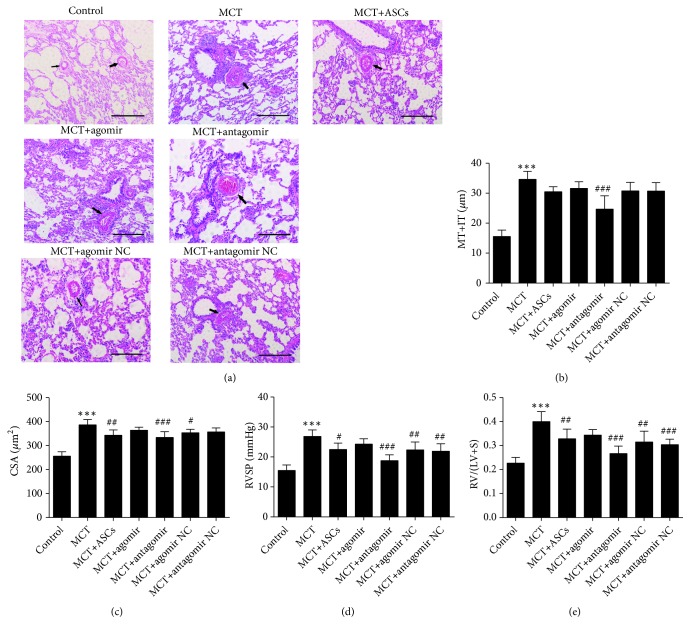
ASCs ameliorated pulmonary and heart function in a rat PAH model. (a) Representative H&E staining pictures of pulmonary artery vascular. Scale bars = 100 *μ*m. (b–e) The comparison of ET+MT, CSA, RVSP, and RV/(LV+S) after different ASCs administration for 2 weeks. Data was presented as the mean ± SD, n =5-6. *∗∗∗*P < 0.001 vs. control group, ^#^P < 0.05, ^##^P < 0.01,^ ###^P < 0.001 vs. MCT group.

ASCs with high or low miR-191 expression were obtained after transfection with miR-191 agomir or antagomir, respectively (Figure 3(a)). Two weeks following MCT administration, ASCs with enriched or diminished miR-191 expression and corresponding negative control (NC) were injected via intraperitoneal vein to PAH rats. Medial wall thickness plus intimal thickness (MT + IT), Cross-sectional area (CSA), RVSP, and right ventricle (RV)-to-left ventricle (LV) + septum (S) (RV/LV + S) values after two weeks of ASCs administration are shown in Figures 4(b)–4(e). RVSP was significantly increased in the MCT group compared with the control group (26.85 ± 1.94 mmHg vs. 15.47 ± 1.69 mmHg; p < 0.001). In contrast, compared with the MCT group, two weeks after ASCs administration, RVSP was notably reduced which was further lowered in the antagomir group (18.77 ± 1.76 mmHg vs. 26.85 ± 1.94 mmHg; p < 0.001). Similar result was observed for RV/(LV+S). The RV/(LV+S) was much higher in the MCT group than in the control group (0.4 ± 0.04 vs. 0.23 ± 0.02; p < 0.001). Nonetheless, two weeks after treatment, RV/(LV+S) significantly decreased in the antagomir group, much lower than the MCT group (0.27 ± 0.03 vs. 0.4 ± 0.04; p < 0.001). To further evaluate the effect of ASCs, right lungs from PAH rats were stained by routine hematoxylin and eosin (H&E) staining (Figure 4(a)). As shown in Figure 4(b), MT+IT was much higher in the MCT group than in the control group (15.54 ± 1.97 *μ*m vs. 34.63 ± 2.41 *μ*m; p < 0.001). Similarly, administration of ASCs significantly decreased MT+IT compared with the MCT group. MT+IT in the miR-191 antagomir group was much lower than the MCT group (24.67 ± 4.04 *μ*m vs. 34.63 ± 2.41 *μ*m; p < 0.001). CSA was significantly increased in the MCT group compared with the control group (386.33 ± 20.12 *μ*m^2^ vs. 256.17 ± 16.80 *μ*m^2^; p < 0.001, Figure 4(c)). ASCs administration, however, reduced CSA. Moreover, CSA in the antagomir group was much lower than in the MCT group (334 ± 21.92 *μ*m^2^ vs. 386.33 ± 20.12 *μ*m^2^; p < 0.001). This evidence suggested miR-191 as a potential risk factor for the development of PAH in rats.

**Figure 5:**
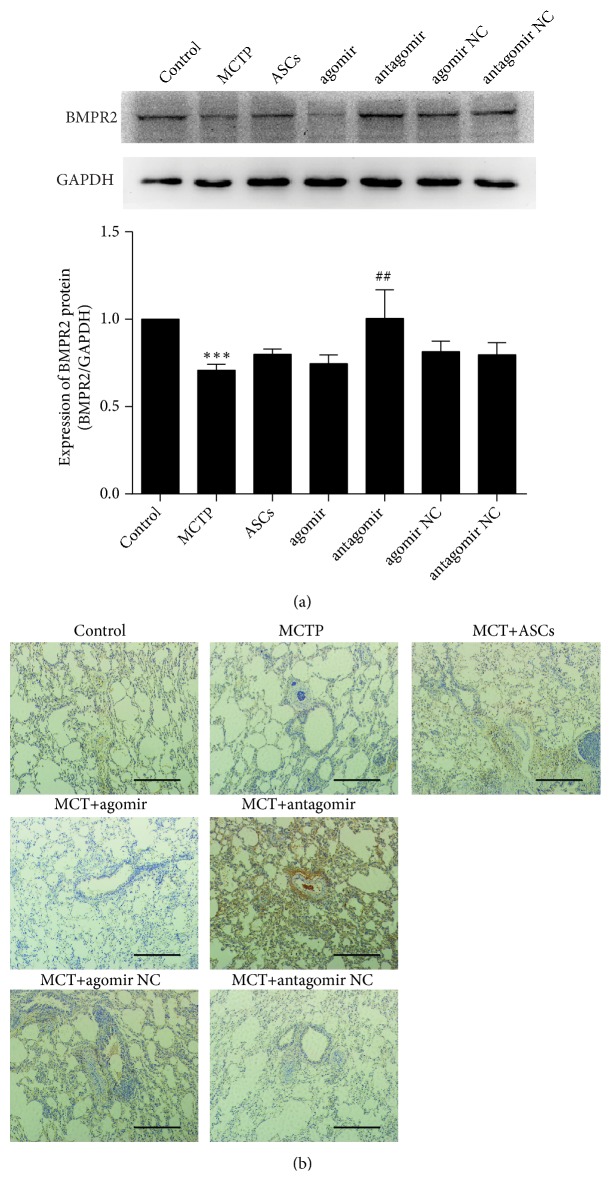
miR-191 directly targets BMPR2 in HPAECs and PAH rats. (a) The protein expression of BMPR2 compared with GAPDH (data is presented as the mean ± SD) in HPAECs. *∗∗∗*P < 0.001 vs. control group, ^##^P < 0.01 vs. MCTP group. (b) Immunohistochemistry staining results for BMPR2 in lungs of rats. Scale bars = 100 *μ*m.

In order to identify downstream targets of miR-191 in PAH rats, we investigated BMPR2 expression in rat lungs. BMPR2 expression was high in the antagomir group and poorly expressed in the MCT group as assessed by immunohistochemistry (Figure 5(b)). On the basis of this result, we found that miR-191 directly affected BMPR2 expression in PAH rats.

## 4. Discussion

Excessive proliferation of HPAECs is one of the major pathological features of vasculature remodeling, and inhibition of excessive proliferation of endothelial cells is a vital element of current PAH therapies [25, 26]. In vitro, we treated HPAECs with MCTP, an alkylating agent that is synthesized from MCT in the liver [27]. Our finding that MCTP inhibited HPAECs proliferation is in line with previous studies reporting that MCTP-induced protein and DNA adducts ultimately were responsible for endothelial cell cycle arrest [28–31]. Next, we explored the effect of ASCs on MCTP-treated HPAECs and observed that ASCs improved proliferation of MCTP-treated HPAECs.

Recently, aberrant expression of miR-191 has been reported to be associated with various cancers (>20) and other diseases including Crohn's, type-2 diabetes, pulmonary hypertension, and Alzheimer's [32]. A recent study has corroborated that miR-191 was one of upregulated miRNAs in the circulation of PH subjects [22]. Interestingly, miR-191 is one of the most represented miRNAs in ASCs-Exos [21]. Compelling reports indicate that exosomes released from ASCs have a therapeutic role through transfer of characteristic proteins, mRNA, as well as miRNAs [33–35]. To elucidate the underlying mechanism of ASCs mediated protection of HPAECs, ASCs-Exos were incubated with MCTP-treated HPAECs. Interestingly, in the presence of the miR-191 antagomir, we observed a significant reversal of protective effect and vice versa with the miR-191 agomir. Thus, these results demonstrated that ASCs-Exos, at least in part, influenced proliferation of HPAECs via miR-191, which subsequently might affect the progression of vasculature remodeling.

PAH is characterized by functional and structural changes in the pulmonary vasculature, and thus it is critical to terminate or reverse the ensuing remodeling of vasculature [3, 9]. ASCs transplantation is considered to be a potential therapeutic choice. Previous studies have shown that ASCs possess the potential to reverse pulmonary arteriole remodeling and hypertrophy of the right ventricle [36–38]. However, the specific mechanisms underlying the ASCs mediated reversal of remodeling are unclear. The MCT-induced rat PAH model, commonly used by researchers, is an inexpensive and reproducible tool that does not require meticulous technical skills [39]. In this study, we utilized this model to explore the influence of miR-191-modified ASCs on PAH. Data showed that miR-191 antagomir induced a significant reversal of vascular remodeling, and downregulation of mediators associated with pulmonary hypertension, suggesting that this effect was, in part, modulated by miR-191. Understanding the mechanisms responsible for miR-191-related vasculature remodeling, thus, may reveal additional strategies for therapeutic intervention.

That BMPR2 signaling plays a critical role in the pathogenesis of PAH, which is evident from previous studies that showed deletion of BMPR2 in endothelial cells induced PAH in mice [40]. Furthermore, overexpression of miR-191 is associated with pulmonary hypertension [22]. Therefore, we explored the involvement of BMPR2 in miR-191 mediated enhancement of growth of HPAECs. Consistently with our previous hypothesis, miR-191 significantly decreased BMPR2 level. In the presence of the miR-191 antagomir, BMPR2 level increased, with corresponding improvement in symptoms of PAH. Therefore, BMPR2 was involved in miR-191-regulated survival of HPAECs.

## 5. Conclusion

Our study showed that inhibition of miR-191 could ameliorate the development of MCT-induced PAH possibly via preventing BMPR2 degradation. Moreover, BMPR2 was involved in miR-191 mediated HPAECs proliferation. Taken together, we found that miR-191 could be a potential risk factor for PAH. Therefore, this study provided the basic insight into the use of anti-miR-191 as a therapeutic strategy against PAH. However, this is a preliminary study to unravel the possible mechanism of miR-191 and its application in PAH which warrants further studies to validate the above findings in patients. This study only illuminates the effect of miR-191 on HPAECs and rat PAH model. The next step would be to explore the exact mechanisms for application in human therapy.

## Figures and Tables

**Table 1:** Sequences of synthesized miR-191 oligonucleotides.

Oligonucleotides	Sequences (5′ to 3′)
miR-191 agomir	CAACGGAAUCCCAAAAGCAGCUG
	CAGCUGCUUUUGGGAUUCCGUUG
miR-191 antagomir	CAGCUGCUUUUGGGAUUCCGUUG
agomir NC	UCACAACCUCCUAGAAAGAGUAGA
	UCUACUCUUUCUAGGAGGUUGUGA
antagomir NC	UCUACUCUUUCUAGGAGGUUGUGA

## Data Availability

The data used to support the findings of this study are available from the corresponding author upon request.
